# Changes in Lipoprotein Fractions and Lipoprotein-Associated Alpha-Tocopherol After Sleeve Gastrectomy in Patients With Severe Obesity

**DOI:** 10.14740/cr2244

**Published:** 2026-07-17

**Authors:** Hisayuki Katsuyama, Yuji Hirowatari, Daisuke Manita, Tatsuya Iwasaki, Sachiyo Yoshio, Susumu Inamine, Mariko Hakoshima, Takumi Kawaguchi, Hidekatsu Yanai

**Affiliations:** a Department of Diabetes, Endocrinology and Metabolism, National Kohnodai Medical Center, Japan Institute for Health Security, Chiba, Japan; b Department of Health Sciences, Saitama Prefectural University, Saitama, Japan; c Bioscience Division, TOSOH Corporation, Kanagawa, Japan; d Department of Human Immunology and Translational Research, National Institute of Global Health and Medicine, Japan Institute for Health Security, Tokyo, Japan; e Bariatric and Metabolic Surgery Center, Ohama Daiichi Hospital, Okinawa, Japan; f Division of Gastroenterology, Department of Medicine, Kurume University School of Medicine, Fukuoka, Japan

**Keywords:** Obesity, Lipoprotein, Sleeve gastrectomy

## Abstract

**Background:**

Sleeve gastrectomy (SG) improves obesity-related metabolic abnormalities, including dyslipidemia. However, postoperative changes in individual lipoprotein fractions and lipoprotein-associated antioxidants remain incompletely understood. This exploratory study aimed to characterize changes in serum lipoprotein fraction profiles after SG in Japanese patients with severe obesity using anion-exchange high-performance liquid chromatography (AEX-HPLC).

**Methods:**

Patients who underwent SG at Ohama Daiichi Hospital between July and December 2020 were enrolled. Clinical data and serum samples were obtained before and after surgery. Serum low-density lipoprotein cholesterol (LDL-C), high-density lipoprotein cholesterol (HDL-C), and triglycerides (TGs) were measured by enzymatic assays, and small dense LDL-C (sdLDL-C) was estimated using Sampson’s equation. Lipoprotein fractions were evaluated by AEX-HPLC. α-tocopherol concentrations in HDL, LDL, and very low-density lipoprotein (VLDL) fractions were measured by reverse-phase high-performance liquid chromatography. Overall changes over time were assessed using the Friedman test, followed by *post hoc* Wilcoxon signed-rank tests with Benjamini-Hochberg false discovery rate adjustment.

**Results:**

Of 20 patients who underwent SG, 12 were analyzed after excluding eight with inadequate stored serum quality. Mean age and body mass index were 45.3 ± 10.5 years and 43.4 ± 5.9 kg/m^2^, respectively. SG significantly reduced body weight, hemoglobin A1c, and enzymatically measured TG, while HDL-C increased significantly. No significant changes were observed in LDL-C or non-HDL-C. Estimated sdLDL-C levels and the sdLDL-C/LDL-C ratio decreased significantly after SG. AEX-HPLC revealed significant increases in HDL and LDL fractions and a significant decrease in the VLDL fraction. α-Tocopherol content in HDL and LDL fractions increased significantly after SG, whereas no significant changes were observed in α-tocopherol-LDL/LDL or α-tocopherol-HDL/HDL. In contrast, α-tocopherol-VLDL/VLDL increased significantly after SG.

**Conclusions:**

SG was associated with improvements in body weight and conventional lipid parameters, including reduced TG and increased HDL-C, together with a reduction in estimated sdLDL-C, in Japanese patients with severe obesity. AEX-HPLC revealed complex postoperative changes in lipoprotein fractions, including reduced VLDL and increased HDL and LDL fractions. Lipoprotein-associated α-tocopherol distribution also changed after SG; however, the clinical significance of these changes requires further investigation.

## Introduction

Obesity is a well-established risk factor for metabolic disorders, including dyslipidemia, insulin resistance, and hypertension, all of which contribute to the development and progression of atherosclerotic cardiovascular disease (CVD) [1, 2]. Obesity-related dyslipidemia is characterized by quantitative and qualitative alterations in plasma lipid metabolism, typically presenting as elevated triglyceride (TG) levels and reduced levels of protective high-density lipoprotein cholesterol (HDL-C) [3].

Bariatric surgery has been recognized as a highly effective treatment for severe obesity, not only achieving significant and sustained weight reduction but also improving metabolic parameters and reducing cardiovascular risks [4]. Sleeve gastrectomy (SG), a restrictive bariatric procedure involving longitudinal resection of the greater curvature of the stomach, has become one of the most widely performed bariatric surgeries because of its relatively low complication rates and favorable metabolic outcomes [5]. Several studies have demonstrated that SG improves glycemic control, blood pressure, and conventional lipid parameters, thereby contributing to reduced cardiovascular risks [6–8]. However, changes in individual lipoprotein fractions have not been consistently characterized, and qualitative changes in lipoprotein metabolism after SG remain incompletely understood. Although postoperative decreases in TG and increases in HDL-C have been consistently reported, findings regarding low-density lipoprotein cholesterol (LDL-C) after SG have been inconsistent across studies [9–11]. This discrepancy may reflect the limitations of conventional lipid measurements. Therefore, a more detailed characterization of lipoprotein fractions is needed to better understand postoperative changes in lipid metabolism after SG.

In addition to quantitative changes in lipoprotein fractions, qualitative changes in the antioxidative properties of circulating lipoproteins may also be associated with cardiovascular risks after SG. Oxidative modification of lipoproteins promotes inflammatory responses within the vascular wall and contributes to the initiation and progression of atherosclerosis; therefore, antioxidant defenses within lipoproteins are essential for maintaining vascular homeostasis [12, 13]. α-Tocopherol is the predominant lipid-soluble antioxidant in circulating lipoproteins and plays a critical role in protecting lipoprotein particles from oxidative modification [14].

Anion-exchange high-performance liquid chromatography (AEX-HPLC) enables detailed quantification of multiple lipoprotein fractions, including HDL, LDL, intermediate-density lipoprotein (IDL), very low-density lipoprotein (VLDL), and a fraction of others including lipoprotein (a) and chylomicron, and can also assess α-tocopherol levels in individual fractions [15–17]. This approach provides a high-resolution evaluation of both lipoprotein distribution and antioxidant capacity beyond conventional lipid assays.

In this exploratory study, we aimed to characterize postoperative changes in serum lipoprotein fraction profiles and lipoprotein-associated α-tocopherol in Japanese patients with severe obesity using by AEX-HPLC, to assess quantitative and qualitative alterations in lipid metabolism following SG.

## Materials and Methods

This study included patients who underwent laparoscopic SG at Ohama Daiichi Hospital between July and December 2020. Clinical data, including anthropometric measurements and biochemical parameters, were collected preoperatively (baseline) and at 6 and 12 months after SG. Serum lipid levels, including total cholesterol (TC), LDL-C, HDL-C, and TG, were measured using enzymatic assays at baseline. TC was determined by the cholesterol oxidase method, TG by an enzymatic colorimetric method, and LDL-C and HDL-C by direct homogeneous assays. In this study, lipoproteins measured by enzymatic assays are denoted as eTC, eHDL-C, eLDL-C, and eTG. Non-HDL cholesterol (non-HDL-C) was calculated as eTC minus eHDL-C. Small dense LDL (sdLDL) concentrations were estimated using Sampson’s equation, a validated indirect method for assessing sdLDL from routine lipid parameter [18].

Residual serum obtained at baseline and at 1, 3, 6, and 12 months after SG was stored at −80 °C and used for subsequent analyses. Patients whose serum samples showed signs of degradation, such as hemolysis or improper storage conditions, were excluded from the analysis to ensure the reliability of lipoprotein and antioxidant measurements.

Lipoprotein profiling was performed using AEX-HPLC, as previously described [15, 16]. In addition, α-tocopherol concentrations in isolated HDL, LDL, and VLDL fractions were measured using reverse-phase high-performance liquid chromatography [17]. This provided a detailed assessment of antioxidant status within each lipoprotein class.

Continuous variables are presented as mean ± standard deviation. Overall changes in parameters over time were assessed using the Friedman test. When the Friedman test indicated a significant overall difference, *post hoc* comparisons between baseline and each postoperative time point were performed using Wilcoxon signed-rank tests. To account for multiple comparisons, P values were adjusted using the Benjamini-Hochberg false discovery rate method. A P-value of < 0.05 was considered statistically significant. All analyses were conducted using SPSS ver. 29.0 (IBM Corp., USA).

This study was approved by the Institutional Review Board for Clinical Research, Japan Institute for Health Security (NCGM-S-003661) and was performed in accordance with the Declaration of Helsinki.

## Results

During the study period, 20 patients underwent SG. Eight patients were excluded because the quality of their stored serum samples was inadequate. Thus, the remaining 12 patients were enrolled for this study. Table 1 shows the characteristics of the patients. The mean age was 45.3 ± 10.5 years, body weight was 111.2 ± 17.6 kg, and body mass index (BMI) was 43.4 ± 5.9 kg/m^2^. Three patients had diabetes, 10 had dyslipidemia, and five had hypertension. Transitional changes in anthropometric measurements and biochemical parameters after SG are presented in Table 2. Body weight decreased by 24.0 kg at 6 months and 25.2 kg at 12 months from baseline. There were also significant reductions in plasma glucose, hemoglobin A1c (HbA1c) and eTG levels, whereas eHDL-C levels increased over time. Serum ALT levels decreased significantly at 12 months. No significant changes were observed in eTC, eLDL-C, or non-HDL-C.

**Table 1:** The Baseline Characteristics of the Patients (n = 12)

Variables	n, mean ± SD
Gender (female/male)	10/2
Age (years)	45.3 ± 10.5
Body height (cm)	159.9 ± 5.5
Body weight (kg)	111.2 ± 17.6
BMI (kg/m^2^)	43.4 ± 5.9
Comorbidities	
Diabetes	3
Dyslipidemia	10
Hypertension	5
Medication	
Antihyperglycemic agents	0
Angiotensin-converting enzyme inhibitor	1
Angiotensin II receptor blockers	4
Calcium channel blockers	4
Statins	3
Ezetimibe	1
Fibrates	2

BMI: body mass index.

**Table 2:** The Postoperative Changes in Metabolic Parameters 6 and 12 Months After Sleeve Gastrectomy (n = 12)

Variables	Baseline	6 months	12 months	Overall P	Adjusted P value	
					6 months vs. baseline	12 months vs. baseline
Body weight (kg)	111.2 ± 17.6	87.2 ± 13.1	86.0 ± 12.6	< 0.001	0.002	0.002
BMI (kg/m^2^)	43.4 ± 5.9	34.1 ± 4.3	33.6 ± 4.4	< 0.001	0.002	0.002
Plasma glucose (mg/dL)	119 ± 29	89 ± 9	90 ± 5	0.008	0.010	0.008
HbA1c (%)	6.2 ± 0.7	5.3 ± 0.2	5.4 ± 0.2	< 0.001	0.004	0.003
AST (IU/L)	34 ± 24	39 ± 67	19 ± 6	0.266	0.270	0.118
ALT (IU/L)	36 ± 26	43 ± 90	17 ± 6	0.008	0.157	0.028
GGTP (IU/L)	59 ± 61	89 ± 168	40 ± 67	0.041	0.533	0.076
eTC (mg/dL)	207 ± 26	212 ± 28	212 ± 18	0.779	0.866	0.754
eHDL-C (mg/dL)	48 ± 8	62 ± 11	65 ± 13	< 0.001	0.003	0.004
eLDL-C (mg/dL)	132 ± 24	128 ± 27	128 ± 24	0.667	1.000	0.556
eNon-HDL-C (mg/dL)	159 ± 24	149 ± 26	147 ± 22	0.338	0.158	0.198
eTG (mg/dL)	194 ± 62	89 ± 35	86 ± 25	< 0.001	0.002	0.002
Creatinine (mg/dL)	0.71 ± 0.15	0.68 ± 0.12	0.65 ± 0.10	0.076	0.145	0.090

Data are expressed as mean ± SD. Overall P values were calculated using the Friedman test. *Post hoc* comparisons with baseline were performed using Wilcoxon signed-rank tests with Benjamini-Hochberg false discovery rate adjustment. ALT: alanine aminotransferase; AST: aspartate aminotransferase; BMI: body mass index; CVD: cardiovascular disease; eHDL-C: high-density lipoprotein-cholesterol measured by enzymatic assays; eLDL-C: low-density lipoprotein-cholesterol measured by enzymatic assays; eTC: total cholesterol measured by enzymatic assays; eTG: triglyceride measured by enzymatic assays; GGTP: gamma-glutamyl transferase; HbA1c: hemoglobin A1c.

**Figure 1:**
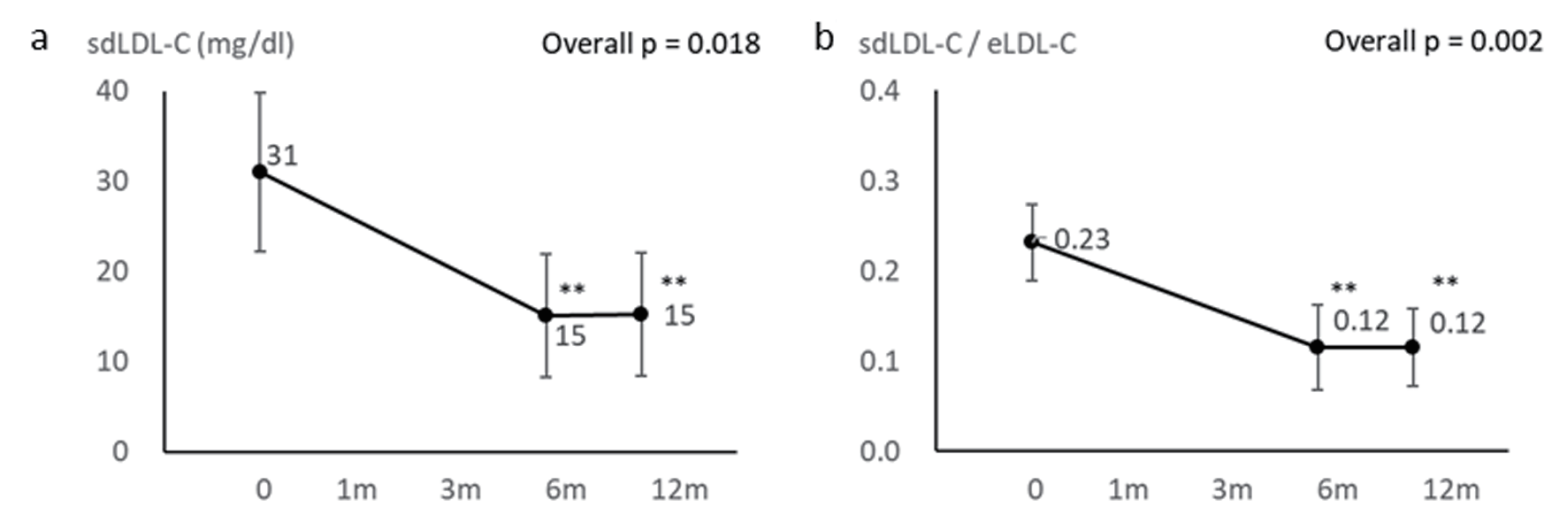
Changes in estimated sdLDL-C levels and estimated sdLDL-C/eLDL-C before and after sleeve gastrectomy. Overall P values were calculated using the Friedman test. *Post hoc* comparisons with baseline were performed using Wilcoxon signed-rank tests with Benjamini-Hochberg false discovery rate adjustment. *Adjusted P < 0.05, **Adjusted P < 0.01 versus baseline. eLDL-C: low-density lipoprotein-cholesterol measured by enzymatic assays; sdLDL-C: small dense low-density lipoprotein cholesterol.

The changes in estimated sdLDL-C levels, calculated by Sampson’s equation, are provided in Figure 1. There were significant decreases in both sdLDL-C levels and sdLDL-C/eLDL-C after SG.

**Figure 2:**
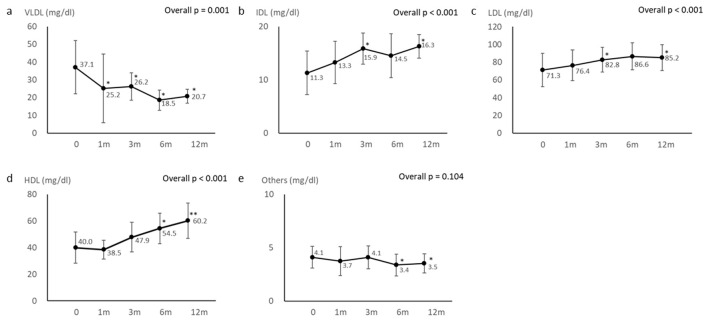
Changes in lipoprotein fractions measured by anion-exchange high-performance liquid chromatography (AEX-HPLC) before and after sleeve gastrectomy. Overall P values were calculated using the Friedman test. *Post hoc* comparisons with baseline were performed using Wilcoxon signed-rank tests with Benjamini-Hochberg false discovery rate adjustment. *Adjusted P < 0.05, **Adjusted P < 0.01 versus baseline.

Longitudinal changes in lipoprotein fractions measured by AEX-HPLC before and after SG are shown in Figure 2. The Friedman test revealed significant overall changes in HDL, LDL, IDL, and VLDL fractions over time, whereas no significant overall change was observed in the fraction of others. In *post hoc* comparisons with baseline using Wilcoxon signed-rank tests with Benjamini-Hochberg adjustment, the HDL fraction was significantly increased at 6 and 12 months after SG. The LDL fraction measured by AEX-HPLC was significantly increased at 3, 6 and 12 months after SG, and the IDL fraction was significantly increased at 3 and 12 months after SG. In contrast, the VLDL fraction was significantly decreased at 1, 3, 6 and 12 months after SG.

**Figure 3:**
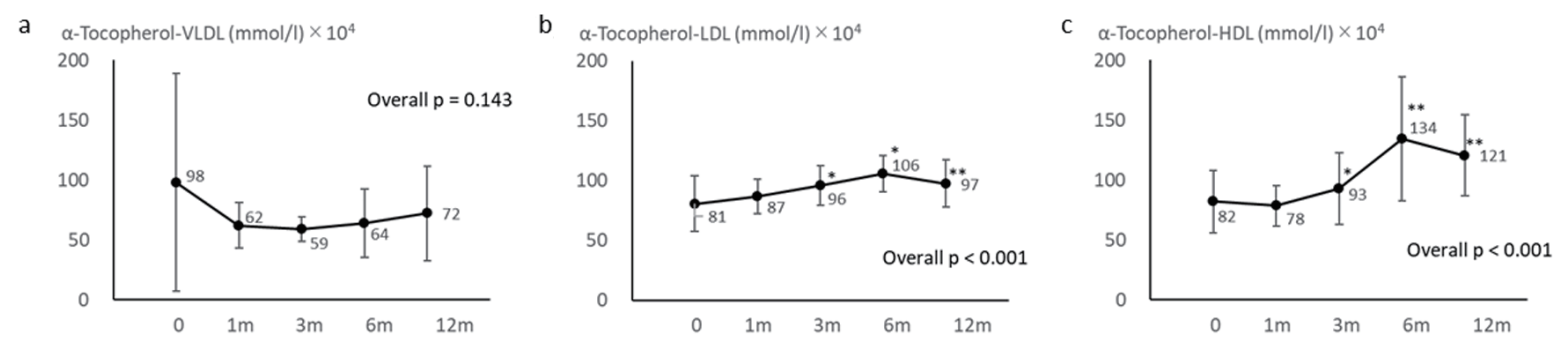
Changes in α-tocopherol content in lipoprotein fractions before and after sleeve gastrectomy. Overall P values were calculated using the Friedman test. *Post hoc* comparisons with baseline were performed using Wilcoxon signed-rank tests with Benjamini-Hochberg false discovery rate adjustment. *Adjusted P < 0.05, **Adjusted P < 0.01 versus baseline.

The longitudinal changes in α-tocopherol content in each lipoprotein fraction before and after SG are shown in Figure 3. The Friedman test revealed significant overall changes in α-tocopherol content in the HDL and LDL fractions over time, whereas no significant overall change was observed in the VLDL fraction. In *post hoc* comparisons, α-tocopherol content in the HDL and LDL fractions was significantly increased at 3, 6, and 12 months after SG.

**Figure 4:**
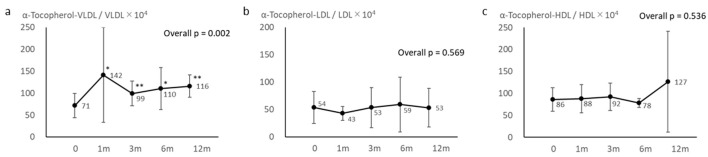
Changes in the α-tocopherol-to-lipoprotein ratio in each fraction before and after sleeve gastrectomy. Overall P values were calculated using the Friedman test. *Post hoc* comparisons with baseline were performed using Wilcoxon signed-rank tests with Benjamini-Hochberg false discovery rate adjustment. *Adjusted P < 0.05, **Adjusted P < 0.01 versus baseline.

Figure 4 shows the post-SG changes in the α-tocopherol-to-lipoprotein ratio in each fraction. The Friedman test revealed a significant overall change in α-tocopherol-VLDL/VLDL over time, whereas no significant overall changes were observed in α-tocopherol-LDL/LDL or α-tocopherol-HDL/HDL. In *post hoc* comparisons, α-tocopherol-VLDL/VLDL was significantly increased at 1, 3, 6, and 12 months after SG.

## Discussion

The present exploratory study demonstrated that SG was associated with significant reductions in body weight, HbA1c, and eTG, together with a significant increase in eHDL-C, while eLDL-C and non-HDL-C remained unchanged in Japanese patients with severe obesity. Estimated sdLDL-C levels and the estimated sdLDL-C/eLDL-C ratio were significantly decreased after SG. AEX-HPLC revealed significant increases in the HDL, LDL, and IDL fractions and a significant decrease in the VLDL fraction. These findings suggest that SG is associated with complex postoperative changes in lipoprotein metabolism that are not fully captured by conventional lipid measurements.

Previous studies have shown that SG is generally associated with reduced TG and increased HDL-C, and our findings are consistent with these observations. In contrast, changes in LDL-C after SG appear less consistent. An Italian observational study reported no significant change in LDL-C at 1 year after SG [8]. Similarly, a meta-analysis of studies with at least 1 year of follow-up and another meta-analysis evaluating the long-term impact of SG reported no significant change in LDL-C [9, 11]. Conversely, a Turkish observational study reported an increase in LDL-C at 6 months and 1 year after SG [10]. Thus, changes in LDL-C after SG may vary across studies. These findings suggest that postoperative lipid changes after SG may not represent a simple uniform improvement, but rather a redistribution of lipoprotein fractions.

In the present study, eLDL-C measured by a direct homogeneous assay did not change significantly after SG, whereas the LDL fraction measured by AEX-HPLC increased. This discrepancy may partly reflect differences in methodology and in the lipoprotein components captured by each assay. Direct LDL-C assays are widely used in clinical practice, but discrepancies from reference methods have been reported, particularly in conditions characterized by abnormal TG-rich lipoprotein metabolism, such as obesity and type 2 diabetes [19–22]. In contrast, AEX-HPLC separately quantifies major lipoprotein fractions, including HDL, LDL, IDL, and VLDL, and may therefore detect changes in lipoprotein distribution that are not fully captured by conventional lipid assays [15, 16]. Nevertheless, the increase in the LDL fraction measured by AEX-HPLC is an important finding, and its clinical significance remains uncertain.

In the present study, VLDL decreased in association with weight loss after SG. Previous detailed lipoprotein analyses also support the concept that SG may induce heterogeneous changes in lipoprotein fractions. In a study using ^1^H-nuclear magnetic resonance to analyze lipoprotein changes before and after SG, VLDL particle number, VLDL cholesterol, and TG decreased, whereas LDL particles and LDL-C increased [23]. Other studies have reported decreases in VLDL after SG in patients with obesity [24], and reductions in apoB100 in association with decreased VLDL [25]. These findings, together with the present results, suggest that SG may be associated with a redistribution of lipoprotein fractions, characterized mainly by reduced TG-rich lipoproteins, rather than a simple decrease in all atherogenic lipoprotein fractions.

The decrease in VLDL observed in the present study may be related to weight loss and improvement in insulin resistance after SG. In severe obesity, insulin resistance enhances hormone-sensitive lipase activity in adipose tissue, thereby promoting TG breakdown and increasing the release of free fatty acids (FFAs), which in turn increases FFA influx to the liver. In the liver, insulin resistance suppresses insulin-mediated degradation of apoB100, resulting in increased production of TG-rich lipoproteins and enhanced VLDL secretion. Furthermore, because lipoprotein lipase activity is reduced in insulin resistance, the catabolism of VLDL and IDL is also impaired. Increased TG-rich VLDL promotes the conversion of LDL to sdLDL and is also associated with reduced HDL levels [26, 27]. Body weight loss and improvement in insulin resistance after SG may have contributed to the decreases in eTG and VLDL and the increase in HDL observed in this study through reduced hepatic VLDL secretion and improved metabolism of TG-rich lipoproteins.

Although the LDL fraction measured by AEX-HPLC increased in this study, estimated sdLDL-C and the estimated sdLDL-C/eLDL-C ratio decreased. sdLDL is known to be highly atherogenic because it is more susceptible to oxidation and more readily penetrates the arterial wall [28]. Therefore, the increase in the LDL fraction observed in this study does not necessarily indicate an increase in atherogenic LDL, but may reflect potentially favorable changes in TG-rich lipoprotein metabolism after SG, characterized by a reduction in TG-rich lipoproteins and a decrease in sdLDL. Indeed, metabolic bariatric surgery, including SG, has been reported to reduce the incidence of CVD compared with non-surgical treatment in patients with obesity and type 2 diabetes [29]. Although the reduction in estimated sdLDL-C may reflect potentially favorable metabolic changes after SG, sdLDL-C was not directly measured but was estimated using Sampson’s equation. In addition, the concomitant increase in the LDL fraction measured by AEX-HPLC makes it difficult to interpret the overall lipoprotein changes as anti-atherogenic. Therefore, the relationship between these findings and cardiovascular risk reduction remains uncertain.

One notable feature of this study is that we evaluated the distribution of α-tocopherol among individual lipoprotein fractions. The absolute amount of α-tocopherol in the HDL and LDL fractions increased after SG, whereas α-tocopherol content in the VLDL fraction did not show a significant change. In contrast, the α-tocopherol-to-lipoprotein ratios in the HDL and LDL fractions did not change significantly, while the α-tocopherol-VLDL/VLDL ratio increased after SG. α-Tocopherol is a major lipid-soluble antioxidant in serum lipoproteins and plays a role in protecting lipoproteins from oxidative modification [14, 30]. The present findings suggest that SG may be associated with postoperative changes in the distribution of lipoprotein-associated α-tocopherol. However, the absolute amount of α-tocopherol in each fraction is influenced by the amount of the lipoprotein fraction itself. Therefore, changes in α-tocopherol levels in the HDL and LDL fractions may partly reflect changes in lipoprotein fraction levels. In contrast, the increase in the α-tocopherol-VLDL/VLDL ratio may reflect a relative change in α-tocopherol content within the VLDL fraction; however, its biological and clinical significance remains uncertain. The present study did not directly measure oxidative stress markers, inflammatory biomarkers, or antioxidant capacity. Therefore, changes in lipoprotein-associated α-tocopherol cannot be interpreted as direct evidence of enhanced antioxidative function. Further studies including direct measurements of oxidative stress and functional assays are needed to clarify the role of fraction-specific changes in α-tocopherol distribution in atherosclerotic risk after SG.

### Limitations

This study has several limitations. First, this was an exploratory study with a small sample size, which limits statistical power and generalizability. Second, this was a single-center study without a control group, making it difficult to clarify causal relationships regarding the effects of SG. Third, the study population was limited to Japanese patients with severe obesity. Dietary patterns, including Japanese dietary habits and postoperative dietary changes, may have influenced lipid metabolism and lipoprotein-associated α-tocopherol distribution. Fourth, the study population had a gender imbalance, and gender-specific analyses could not be performed. Fifth, several patients were receiving lipid-lowering medications, including statins, fibrates, and ezetimibe, which may have affected lipid profiles and lipoprotein fractions. Because of the small sample size and the limited number of patients receiving each medication, meaningful subgroup analyses according to medication class were not feasible. Sixth, the storage conditions of the serum samples may have influenced the measurement results. Seventh, eight patients were excluded because of inadequate stored serum quality, which may have introduced selection bias. Eighth, estimated sdLDL-C was calculated using Sampson’s equation rather than directly measured. Finally, the study did not assess oxidative stress markers, inflammatory biomarkers, and antioxidative capacity. Future larger controlled studies with direct assessment of oxidative stress, dietary intake, medication effects, and longer follow-up are needed to confirm the clinical significance of our findings.

### Conclusions

SG was associated with reductions in body weight and improvements in conventional lipid parameters, including reduced TG and increased HDL-C, together with a reduction in estimated sdLDL-C, in Japanese patients with severe obesity. AEX-HPLC analysis showed postoperative changes in lipoprotein fractions, including decreased VLDL and increased HDL and LDL fractions. Changes in the distribution of lipoprotein-associated α-tocopherol were also observed, although their biological and clinical significance remains uncertain. Further studies are needed to clarify the clinical implications of these lipoprotein and antioxidant-related changes after SG.

## Data Availability

The authors declare that data supporting the findings of this study are available within the article.

## Abbreviations

AEX-HPLC: anion-exchange high-performance liquid chromatography
ALT: alanine aminotransferase
AST: aspartate aminotransferase
BMI: body mass index
CVD: cardiovascular disease
eHDL-C: high-density lipoprotein-cholesterol measured by enzymatic assays
eLDL-C: low-density lipoprotein-cholesterol measured by enzymatic assays
eTC: total cholesterol measured by enzymatic assays
eTG: triglyceride measured by enzymatic assays
GGTP: gamma-glutamyl transferase
HbA1c: hemoglobin A1c
HDL-C: high-density lipoprotein cholesterol
LDL-C: low-density lipoprotein cholesterol
sdLDL-C: small dense low-density lipoprotein cholesterol
SG: sleeve gastrectomy
TG: triglyceride
VLDL: very low-density lipoprotein
